# Self-Regulation of Slippery Deadlines: The Role of Procrastination in Work Performance

**DOI:** 10.3389/fpsyg.2021.783789

**Published:** 2022-01-06

**Authors:** Piers Steel, Daphne Taras, Allen Ponak, John Kammeyer-Mueller

**Affiliations:** ^1^Organizational Behaviour and Human Resources, University of Calgary, Calgary, AB, Canada; ^2^Ted Rogers School of Management, Ryerson University, Toronto, ON, Canada; ^3^Haskayne School of Business, National Academy of Arbitrators, University of Calgary, Calgary, AB, Canada; ^4^Carlson School of Management, University of Minnesota, Minneapolis, MN, United States

**Keywords:** procrastination, motivation, dynamic, time, delay, arbitration

## Abstract

We investigated the causes and impact of procrastination on “slippery deadlines,” where the due date is ill-defined and can be autonomously extended, using the unique applied setting of grievance arbitration across two studies. In Study One, using 3 years of observed performance data derived from Canadian arbitration cases and a survey of leading arbitrators, we examined the effect of individual differences, self-regulatory skills, workloads and task characteristics on time delay. Observed delay here is a critical criterion, where justice is emphasized to be *swift* and sure. Multilevel Modeling established trait procrastination as a substantive predictor of observed delay, equivalent to the environmental contributors of expediting the arbitration procedure or grievance complexity. Also, despite substantive negative consequence of delay for both arbitrators and their clients, arbitrators who scored one standard deviation above the mean in procrastination took approximately 83 days to write their decisions compared to the 26 days for arbitrators one standard deviation below the mean. In Study Two, we conducted a replication and extension survey with a much larger group of American arbitrators. Consistent with Temporal Motivation Theory (TMT), trait procrastination was largely explained by expectancy, value, and sensitivity to time related traits and skills, which together accounted for majority of the variance in trait procrastination, leaving little left for other explanations. For example, perfectionism connection to procrastination appears to be distal, being largely mediated by each of TMT’s core variables. Finally, procrastination was largely synonymous with a deadline pacing style, indicating that observed delay can be used as a proxy for procrastination as long as little or no prior work was done (e.g., a u-shaped pacing style is not synonymous). In all, our results indicate that procrastination is rampant in the workplace and has seriously detrimental effects.

## Introduction

Procrastination is an enduring vice, with descriptions in ancient Egypt and Greece dating to the invention of the written word (Benderitter, 2011). Today, procrastination is endemic, with chronic procrastination rising from 5% of the population in the 1970s to approximately 30% in 2010 (Steel and Ferrari, 2013), that is scoring four or higher on a five-point scale. This is notable because procrastination is inherently an irrational or a self-defeating behavior. By definition, we procrastinate when we voluntarily put off until later what we think we should be doing now despite expecting the worse for our delay. More broadly, it reflects an intention-action gap, were we leave actions past their optimal starting date (van Hooft et al., 2005). A review by Steel (2011) confirms what procrastinators suspected, that the ramifications of their delays are indeed typically negative, from health to wealth to happiness. For example, the average income difference between those who report minimal versus maximal procrastination approaches $60,000 per year (Nguyen et al., 2013). Similar, patients failing to comply with medical advice has long been a serious issue, with procrastination singled out as a significant contributor (Becker and Maiman, 1975). Reflecting its deleterious impact and increased prevalence, Surowiecki (2010) notes that “the study of procrastination has become a significant field in academia, with philosophers, psychologists, and economists all weighing in” (p. 110).

Despite such prominence, as Sonnentag (2012) summarizes, “Time should be an important aspect of organizational theory, but it has been neglected for decades” (p. 1). This is expected as understanding why we procrastinate has been hampered by three methodological obstacles. First, there is choice of sample. Workplace procrastination is rife, with estimates that approximately a quarter of most people’s working day can be characterized as procrastinating, with attendant productivity costs (D’Abate and Eddy, 2007; Steel, 2011). Yet, over 90% of studies are conducted with student samples and only about 1% focuses on employe samples (Steel, 2007). Student samples have provided rich data, with daily diary studies indicating students spend a third of their day putting off tasks (Pychyl et al., 2000), but the results do not always generalize to an adult working population (Sackett and Larson, 1990). In particular, the Attraction-Selection-Attrition (ASA) model indicates that those who procrastinate excessively could either not select, not be selected, or simply leave positions where their dillydallying is detrimental. Nguyen et al. (2013) work on job characteristics and procrastination supports this that is “jobs that require higher levels of motivational skills are less likely to retain procrastinators” (p. 388). Consequently, procrastination may not be as relevant to employers as it is to educators.

Second, the research in this area is predominantly mono-method, based almost exclusively on self-reports with only a few notable exceptions (e.g., Elvers et al., 2003; Reuben et al., 2015). To some extent, this reflects what is needed to operationalize the construct; since procrastination is an irrational delay according to one’s own standards, it can only be inferred by an observer. Generally lacking is research that makes use of objective measures that can be examined alongside self-report measures. Furthermore, most of this research is concurrent instead of longitudinal, with the latter really required to assess the effects of delay. Even when it does rarely occur, as Roe (2014) reviews, motivational longitudinal research is often compromised, typically sampling only two or three time points for a short task. Instead, Roe suggests that we should deeply assess longer term projects favoring numerous time points over number of people, preferably all of whom are pursuing a similar goal. So far, only Steel et al. (2018) have managed to address all these specifications, including using an observed measure of delay, but still in an educational setting being based on a computerized Personal System of Instruction.

Third, most studies have focused on tasks with hard deadlines, including Steel et al. (2018), that is those with fixed due dates. Exams, essays and other assignments in academic courses predominantly use hard deadlines, with procrastinators doing an increasing amount of work as these deadlines approach. Business settings in contrast can have “slippery deadlines,” where task completion is not fixed and can be autonomously extended further into the future. For example, where exams and essays often have unnegotiable deadlines, projects completion dates may be unspecified. It is unclear how hard deadlines compare to slippery ones. The dominant explanation of procrastination is temporal discounting (Steel, 2007, 2011), resulting in most work done when delay becomes small (i.e., before a deadline). With a slippery deadline, delay never necessarily diminishes and consequently the effects of temporal discounting is uncertain.

Due to this uncertainty, we do not know exactly how procrastination manifests in the workplace, particularly those with slippery deadlines. Previous reviews by Steel (2007, 2011) stress the importance of individual difference variables of impulsiveness, expectancy and value for predicting procrastination, but the degree they generalize to the workplace is unknown. As Nguyen et al. (2013) discuss, the workplace is often considered a strong environment which diminishes the strength of individual differences. Furthermore, employes may be selected or select themselves for the work environment, resulting in procrastination occurring typically in low-cost situations. For example, having intrinsic motivation for a task potentially eliminates the negative impact of impulsiveness as the reward is experienced while completing the task itself.

### The Labor Arbitration Setting

To further investigate procrastination, we sought a Natural Decision Making (NDM) setting (Klein, 2008). As reviewed by Lipshitz et al. (2001), “NDM is an attempt to understand how people make decisions in real-world contexts that are meaningful and familiar to them” (p. 332). The NDM approach is descriptive (i.e., how people actually make decisions) rather than normative (i.e., a rational or ideal decision-making model) and, by studying people in their “natural habitat,” it tests the degree to which laboratory findings generalize and are practically applicable, that is *external validity*. This approach also addresses the criticism that the applied psychological or organizational behavior field has a dearth of field research (Cialdini, 2009), with Baumeister et al. (2007) direct call for “a renewed commitment to including direct observation of behavior whenever possible and in at least a healthy minority of research projects” (p. 396). Ideally, in this setting individuals have autonomy over their work tasks, style, and pace. There must be a stock and flow of tasks needing attention, with incentives for completion of work. The dependent variable of time must be unobtrusively observed and accurately measured, with some consistency in task from person to person, and from product to product.

To this end, arbitrators are the ideal subjects. Labor arbitration is a widely adopted procedure to resolve disagreements about the application and interpretation of collective bargaining agreements (CBA) between unionized workers and their employers as well as the appropriateness of employe discipline (Bemmels, 1988; Bemmels and Foley, 1996; Trudeau, 2002; Ruben, 2003; Thornicroft, 2010). Under the model used in United States and Canada, employes who believe that a contractual term of their CBA has been violated can file a grievance alleging a contract violation and seeking a remedy. Most grievances are then subject to joint union-management discussion involving progressively higher levels of the respective organizations and most are resolved during such discussion (Lewin, 1999). Unresolved grievances are subject to arbitration.

Consequently, arbitrators produce written decisions that bring justice into the workplace, often juggling multiple cases while maintaining a regular pipeline of appointments. They must accept new cases while urgently trying to find time and energy to issue awards well past the dates of the actual hearings. Potentially, with arbitrators we can consider all three major sources of variability that could influence decision time: task characteristics, the work environment, and individual differences. Of particular interest is whether these individual difference variables remain relevant when taken out of the laboratory and examine in a NDM setting. We review each of these contributions in turn.

In an empirical study of labor arbitration cases using event history analysis, Ponak et al. (1996) divided the labor arbitration process into four distinct stages: (1) pre-arbitration grievance steps; (2) arbitrator selection; (3) hearing scheduling; and (4) preparation of the arbitration award (which we will refer to as “Decision Time”). This final stage accounts for approximately 20 percent of the overall elapsed time from the filing of the grievance to the arbitrator’s decision (Steiber et al., 1985; Ponak and Olson, 1992). The first three stages are subject to the input of multiple parties, often with competing interests. The final stage, however, is almost entirely controllable by arbitrators and they have considerable discretion over Decision Time. Here, their autonomy is among the highest for any professional. They customarily work independently, have a great deal of control over their work scheduling, and over how to balance their work and private commitments. There are few deadlines except ones self-imposed, that is ‘‘slippery deadlines’’ in that they can be pushed ever forward into the future^1^. This makes their work environment not so strong that there isn’t room for discretionary behavior (Mischel, 1977; Withey et al., 2005).

On the other hand, unnecessary delay is strongly discouraged. A body of literature exists on the sources of arbitration delay and there is a consensus that delay is increasing and is harming the labor relations system (Berkeley, 1989; Ponak and Olson, 1992; Thornicroft, 1995; Foisy, 1998; Lewin, 1999; Trudeau, 2002). More colorfully titled articles along this line include “The Well-Aged Arbitration Case” (Ross, 1958) and “Delay: The Asp in the Bosom of Arbitration” (Seitz, 1981). The parties to the arbitration anxiously await the decision, being consequential to grievances and to union-management relations. Furthermore, arbitrators feel an obligation to dispense justice quickly, being well aware of the adage “justice delayed is justice denied.” An arbitrator’s future acceptability may be harmed by a reputation for tardiness (Berkeley, 1989). There also is a financial incentive for arbitrators to work quickly toward a decision because the issuance of an award allows final billing to the parties. The National Academy of Arbitrators (NAA), the pre-eminent professional association of North American labor arbitrators, contains a section in its Code of Professional Responsibility devoted to “Avoidance of Delay” and arbitrators have been sanctioned for undue delay. Clearly, undue delay by an arbitrator is irrational.

A summary of arbitrator Decision Time reported averages ranging from 37 to 101 days depending on the time period covered and region (Thornicroft, 2010). These averages undoubtedly mask considerable variation from case to case, but most studies either report averages and medians without other statistics, or do not separate Decision Time from other parts of the process. The most comprehensive study of Decision Time and its predictors analyzed 500 arbitration decisions over a 4-year time period in the province of Alberta. It found a mean Decision Time of 65 days, a standard deviation of 56 days, and a maximum Decision Time of almost 1 year (Ponak et al., 1996). Notably, all previous studies that sought to explain Decision Time were based on content analysis of written awards, and scholars were able to explain less than one-third of the variance in Decision Time.

With the assistance of two co-authors, who are themselves highly placed within the arbitration community, we were able to comprehensively assess two groups of arbitrators, one Canadian and one American. Though the American sample has the advantage of being larger in size, in Canada, the law requires that all arbitration decisions be publicly reported and are available electronically. Critically, this reporting stipulation allows the Canadian sample to provide an unobtrusive measure of delay but also allows it to almost fully meet all of Roe (2014) suggested criteria for longitudinal studies (e.g., extended time, numerous points of assessment, common task). All data and analyses are available in an Open Science archive: https://osf.io/qsfht/?view_only=3a2144fa0d9d4a388197e33caddc825b.

## Theoretical Causes of Arbitrators’ Delay and Procrastination

Given that this venue is uniquely suited to assessing procrastination with an *extremely* difficult to access sample of professional arbitrators whose individual differences have not been assessed before, making further study unlikely, we sought to comprehensively explore why delays occur. To this end, we considered broadly traits, self-regulatory skills, and situational influences that might account for the unexplained variance within each of our arbitration samples. As mentioned, the smaller Canadian sample is uniquely suited for depth (i.e., investigating tasks with multi-level modeling) and the larger American sample allows for cross validation and expansion of correlation findings.

### Dispositional Traits Influencing Delay

Decision Time should be partially accounted for by motivational individual difference factors. Even when people have the capability, opportunity and intention for action, self-imposed delays can occur. Studied under a variety of terms depending upon the field, such as an “intention-behavior gap” (Sheeran, 2002), “value-action gap” (Barr, 2004) and “attitude-behavior consistency” (Crano and Prislin, 2006), people procrastinate due to individual differences, with procrastination itself appearing to be a personality trait as well. Enduring across time and situation, self-report procrastination has an average test-retest reliability after 42 days of 0.73 (Steel, 2007) and, like other personality traits, approximately 50% of the variance in trait procrastination is inheritable (Luciano et al., 2006). Here, we consider procrastination, expectancy, value, sensitivity to time, and perfectionism.

#### Procrastination

Arbitrators who score high on a trait procrastination scale should take longer to render their decisions. To explain why people procrastinate, Steel (2007) meta-analytic work found three major factors accounting for most of the variance: expectancy, value and time. These three factors form the basis of Temporal Motivational Theory (TMT), which has been applied specifically to procrastination. Accordingly, as *Expectancy* of success and the *Value* of the outcome increases, so does motivation. On the other hand, *Sensitivity to Time* creates hyperbolic discounting, in that the longer an outcome is delayed, the less our motivation. Those who are more sensitive to the effects of time, are distractible or have difficulty delaying gratification, tend to procrastinate more.

*Hypothesis 1.1*: Arbitration decision time should be positively associated with trait procrastination.

*Hypothesis 1.2*: Trait procrastination should be predominantly (i.e., at least 50% of the variance) predicted by expectancy, value, and sensitivity to time variables.

*Hypothesis 1.3*: Trait procrastination should mediate the expectancy, value, and sensitivity to time variables relationships with arbitration decision time.

#### Expectancy

Expectancy refers to self-confidence or self-efficacy that is the degree to which we believe we can successfully complete a task. Numerous reviews confirm that the positive relationship between self-efficacy and performance holds true across a wide variety of settings and occupations (e.g., Bandura, 1997; Stajkovic and Luthans, 1998). Self-efficacy increases performance by influencing goal choice, how long people persevere in the face of difficulties and setback, and the intensity of goal pursuit. It also moderates the relationship between goal planning and goal behavior (Lippke et al., 2009). Consequently, those with lower self-efficacy are less likely to choose to work, will work at a more lackadaisical pace, and are more likely to give up once they encounter a scheduling conflict or other obstacles. Accordingly, Steel (2007) meta-analysis found a negative correlation of −0.46 based on 39 studies between self-efficacy and procrastination. We expect that arbitrators who doubt their abilities are more likely to delay.

*Hypothesis 2.1*: Arbitration decision time should be negatively associated with trait self-efficacy.

#### Value

Showing a similar strength and direction as expectancy or self-efficacy, value decreases the likelihood of procrastination. Value refers to the reward or pleasure we get from completing or conducting a task. As Steel (2007) meta-analysis confirms, we tend to put off tasks that we find aversive. Consequently, arbitrators who dislike writing, a major component of creating a decision, are expected to delay. In addition, the meta-analysis found that one of the top reasons people give for procrastination is “Didn’t have enough energy to begin the task,” linking it with task aversiveness. The connection between low energy and aversiveness is well established, with positive affect itself defined as “a state of high energy, full concentration, and pleasurable engagement” (Watson et al., 1988; p. 1063).

Furthermore, there is also a reliable relationship between low affect or energy and reduced self-regulatory skills. Wagner et al. (2012), drawing on the ego depletion model of self-regulation, found that those who lacked sleep are more likely to “cyber loaf.” Similarly, in a series of studies, Tice et al. (2007) found that increasing or restoring positive mood result in increased self-regulatory strength. Directly investigating this, Gröpel and Steel (2008) report that energy is linked with enthusiasm and pleasurable engagement, finding that chronic *lack of energy*, that is at a trait level, is one of the best predictors of procrastination. Along these lines, repeatedly research has connected bedtime procrastination to reduced energy and decreased self-regulation, which itself results in more bedtime procrastination (e.g., Exelmans and Van den Bulck, 2021). Accordingly, arbitrators lower in trait energy should take longer to issue awards.

Finally, need for achievement represents an individual’s desire for significant accomplishment, mastering of skills, or high standards. A reliable predictor of performance (Judge and Ilies, 2002), those with a higher need for achievement tend to reap more pleasure from accomplishment and often strive for the recognition of their achievements. Steel (2007) meta-analysis, based on 17 studies, places the disattenuated correlation between need for achievement and procrastination at −0.55, making it one of the stronger predictors. For arbitrators, the release of a decision may be a proxy for such recognition or perhaps timely decision releases may be deemed critical for a “successful” arbitrator.

*Hypothesis 3.1*: Arbitration decision time should be positively associated with task aversiveness.

*Hypothesis 3.2*: Arbitration decision time should be positively associated with lack of energy.

*Hypothesis 3.3*: Arbitration decision time should be negatively associated with need for achievement.

#### Sensitivity to Time

As Steel (2007) notes, several variables are associated with sensitivity to delay or sensitivity to time, including “distractibility, impulsiveness, and self-control” (p. 73), with Sharma et al. (2014) meta-analytic principal-components factor analysis indicate they all come under a Disinhibition versus Constraint/Conscientiousness factor. Collectively, they are among the strongest predictors of procrastination, meta-analytically demonstrating an absolute disattentuated correlation of 0.62 and account for 100% of the genotypic variance (Gustavson et al., 2014). They enable procrastination by hindering people’s ability to delay gratification and to work on what is presently difficult and aversive. Alternatively, taking a behavioral economics approach, Ross et al. (2010) describe them as enabling “hot preference for viscerally attractive awards that operate on agents with disproportionate strength at short ranges” (p. 2). When you are susceptible to temptation, you focus attention upon desires of the moment, neglecting or ignoring long-term responsibilities (e.g., Kuhl, 2000).

*Hypothesis 4.1*: Arbitration decision time should be positively associated with distractibility.

*Hypothesis 4.2*: Arbitration decision time should be positively associated with susceptibility to temptation.

#### Perfectionism

Perfectionism has proved a controversial topic for procrastination, with early clinical efforts indicating that this is a major cause but later correlational research suggesting the relationship is weak, negligible or illusionary (i.e., due to an associated third variable). Part of the debate depends on the subdimension focused upon, especially since perfectionism can include the “Organization” construct, which has large and consistently negative correlations with procrastination (Steel, 2007). Including it can mask other positive relations. Two meta-analyses have focused on perfectionism and procrastination (Sirois et al., 2017; Xie et al., 2018), both drawing on Stoeber and Otto (2006) distinction between perfectionist concerns and perfectionist strivings. Perfectionist concerns involve focusing on being evaluated, including perceived discrepancies between performance and standards. Perfection strivings is having high personal standards or expectations. Consistently, perfectionist concerns are considered maladaptive (especially concern with other’s evaluation of oneself) while perfectionist strivings were more adaptive (especially striving for high personal standards). As per Sirois et al.’s meta-analysis, the former generated a positive correlation of 0.231 and the latter a negative correlation of −0.218, with similar results by Xie et al. (2018).

However, Sirois et al. (2017) noted that “the differential associations of procrastination to multidimensional perfectionism may also be due to underlying levels of self-efficacy and impulsivity” (p. 154). Xie et al. (2018) partially investigate this, finding that self-efficacy fully mediates the relationship between perfectionism and procrastination, concluding that “TMT can account for the link between perfectionism and procrastination” (p. 404). We replicate and extend the analyses, assessing whether our other predictors also mediate the relationship between procrastination and other-oriented perfectionist concerns, particularly discrepancies.

*Hypothesis 5.1*: Perfectionism discrepancy should be weakly associated with procrastination.

*Hypothesis 5.2*: Perfectionism discrepancy association with procrastination should be mediated by multiple individual difference variables, especially those related to self-efficacy and sensitivity to time.

### Self-Regulatory Skills Influencing Delay

Hoyle (2010) reviews several frameworks that integrate self-regulation strategies with personality traits. One way is to consider self-regulation as the proximal outcome of distal personality processes. From this perspective, there is a loose relationship between the two, providing room for the independent acquisition of self-regulatory strategies and for them to account for unique variance. For example, though organizational skills are related to the personality trait of conscientiousness, they can be trained and acquired (Klein and Lee, 2006). With regards to procrastination, Koch and Kleinmann (2002) argue that time management skills should help reduce hyperbolic discounting and impulsiveness. Using TMT (Steel and König, 2006), we explore three strategies that should help, hinder or reflect arbitrator delay beyond what personality can explain: Organization, Multitasking and Pacing Style.

#### Organization

Procrastinators tend to be disorganized or, as Schouwenburg (2004) puts it, suffering from “lack of work discipline, lack of time management skill, and the inability to work methodically” (p. 8). Indeed, the disattenuated meta-analytic correlation between organization and procrastination is −0.45 (Steel, 2007). Being organized enhances the ability to set proximate goals, which dependably increases motivation (Locke and Latham, 2004). This effect directly follows from shortening the Delay variable in TMT (Steel and König, 2006; Steel and Weinhardt, 2018). For example, Renn et al. (2011) as well as Gröpel and Steel (2008) confirmed the negative relationship that procrastination has with goal setting, organizing and other forms of self-management. As a result, organized arbitrators are anticipated to have less Decision Time than those whose arbitration practice is more chaotic.

*Hypothesis 6.1*: Organization should be negatively associated with arbitration decision time.

*Hypothesis 6.2*: Organization should be negatively associated with procrastination.

*Hypothesis 6.3*: Organization should partially mediate the relationship between procrastination and arbitration decision time.

#### Multitasking

Multitasking is a form of polychronicity, where we work on several tasks at once or quickly move among multiple tasks. As a work strategy, it is typically but not entirely negative. Those with higher levels of working memory and fluid intelligence, who are dealing with simpler and less cognitively taxing tasks, can indeed multitask effectively (König et al., 2005; Sanderson et al., 2013). However, people can *impulsively* choose to multitask despite performance decrements because it is pleasurable; novelty is rewarding and we acquire this as we switch our attention (König and Waller, 2010; König et al., 2010). Consequently, multitasking can be motivated by susceptibility to temptation, where we switch our attention simply because it is immediately rewarding (Schnauber-Stockmann et al., 2018). On balance, we expect that multitasking is associated with higher levels of procrastination and susceptibility to temptation.

*Hypothesis 7.1*: Multitasking should be positively associated with arbitration decision time.

*Hypothesis 7.2*: Multitasking should be positively associated with procrastination.

*Hypothesis 7.3*: Multitasking should be positively associated with susceptibility to temptation.

*Hypothesis 7.4*: Multitasking should partially mediate the relationship between susceptibility to temptation and procrastination.

#### Pacing Style

As reviewed by Steel et al. (2018), pacing refers to how work processes are spread out over time. For procrastinators, it is almost definitional that they take a deadline approach, completing the bulk of their work at the end, with strong empirical support for this style as well. However, delaying behavior alone may not be procrastination and some may delay due to rational reasons, perhaps adeptly making use of the increased focus that occurs before the deadline. For example, Gevers et al. (2015) argue that though procrastination and pacing style overlap, the two are sufficiently different that there should be incremental variance by the latter. To further validate using delay alone as a proxy of procrastination, we examine the relationship between a deadline pacing style and procrastination.

*Hypothesis 8.1*: A deadline pacing style should be almost synonymous with procrastination.

### Task Environment Influencing Delay

Though all procrastination requires delay, not all delay is procrastination. People may put off finishing a task due to other more pressing obligations or a task may be more onerous and take longer. Previous arbitration studies on task delay have exclusively focused on the work or task environment (e.g., Rose, 1986; Barnacle, 1991; Ponak et al., 1996), overlooking or discounting the impact of dispositional variables and self-regulatory techniques. Still, both the type and the process of grievance did influence time delay, accounting for up to 27% of the variance (Thornicroft, 1995). Drawing on these past examinations, we re-examine several key task or environmental factors. In particular, we are interested in comparing the power of these traditional external predictors of delay with individual difference variables.

#### Workload

It is often suggested that arbitrators, as a group, are “heavily over-committed” (Seitz, 1981), and as a result, arbitrators with a heavier workload will be slower to release decisions. However, Claessens et al. (2010) did not find that workload was related to work completion for a group of R&D engineers. Furthermore, as per Ponak et al. (1996) arbitration study, they found the opposite was true; arbitrators with heavy workloads tend to delay less, perhaps reflecting the Benjamin Franklin’s adage ‘‘If you want something done, ask a busy person.’’ Workload can be operationalized in terms of the observed number of publicly available written arbitration decisions the arbitrator released in the given year^2^. We disagree with this line of research using observed workload.

The appropriateness of using actual observed workload in this context is debatable. As Berry (1990) wrote, “Time diaries may be the most accurate way to measure how people actually spend their time, but it is the perception that shapes behavior. People who believe they are pressed for time will respond according” (p. 32). Accordingly, workload is often studied using retrospective accounts of time use under the term *busyness*. As defined by Gershuny (2005), “Busyness plainly relates to externally observable work or leisure activities, but the state itself is entirely subjective” (p. 287). Consequently, it can be captured in an arbitration setting through self-reported professional commitments (e.g., mentoring or training other arbitrators, participating in conferences, sitting on professional boards or committees) and personal commitments (e.g., child-rearing responsibilities, illness, divorce, or eldercare issues). Previous research has primarily focused on research and academic writing, finding a positive relationship between delay and perceived busyness (e.g., Boice, 1989; Thompson et al., 2008), but Jiraporn et al. (2009) also found this negative relationship between busyness and performance for corporate directors as well. This is consistent with Hockey (2011) resource allocation model that indicates there is a limit to which increased workload can be offset through increased effort.

Consequently, we expect that self-reported workload, which captures more of life’s commitments and potential work-family conflict, should lead to more delays but not necessarily irrational delays that is procrastination. Procrastination is delaying without good reason, with competing responsibilities potentially providing legitimate alternatives.

*Hypothesis 9.1*: Self-reported workload should be positively associated with arbitration decision time.

#### Expedited Grievances

Some expedited processes are established by mutual agreement of the parties (e.g., Canadian Railway Office of Arbitration, Canada Post), or imposed by statute (e.g., Ontario, British Columbia). Although individual schemes vary, expedited procedures often include speeding the process such as by skipping steps or reducing the time allowed to select arbitrators, schedule hearings, and issue awards. Consequently, expedited arbitration provides processes designed to shorten the time to decision.

*Hypothesis 10.1*: Expedited grievances should be negatively associated arbitration decision time.

#### Grievance Complexity

Generally, only the most thorny and significant grievances cannot be resolved internally and proceed to arbitration (Trudeau, 2002). There has been a marked growth in the complexity of the law that must be considered in many decisions. For example, while anti-discrimination rules or certain legislated employment standards may not be expressly set out in the collective agreement, the arbitrator is obliged to respect their provisions and interpret the collective agreement to conform to employment related statutes (Weber v Ontario Hydro, 1995; *Parry Sound*). Sensibly, studies have found that Decision Time in a simple case is less than a complicated one (Ponak et al., 1996). Complexity is usually operationalized by page length of the decision (i.e., complex grievance require more pages to be addressed) and time span of hearing days (i.e., due to the complexity of the grievance, parties will often require scheduling of additional hearing days).

In addition to complex grievances taking longer, we also expect an interaction between grievance complexity and procrastination. When examining deadline driven goal striving, Schmidt et al. (2009) found *interactions* between the individual difference variable of goal orientation and the environment. Under certain conditions, those with a strong avoidance orientation tend to focus on more readily attainable tasks. Similarly, procrastination tends to increase under a variety of conditions, especially when the task becomes more difficult, when it becomes less enjoyable or when the time to completion increases (Steel, 2007). In the words of Lay and Brokenshire (1997), “procrastinators are more susceptible to variation in task aversiveness, compared to non-procrastinators” (p. 85), with susceptibility necessarily indicating an interaction. Complex tasks potentially have all three elements: difficult, less enjoyable and lengthy. Though everyone should take longer finishing complex grievances, procrastinators should take even longer than usual.

*Hypothesis 11.1*: Grievance complexity should be positively associated with arbitration decision time.

*Hypothesis 11.2*: The interaction between grievance complexity and procrastination should be positively associated with arbitration decision time.

## Materials and Methods

With a few notable differences, our method of investigation is similar to Claessens et al. (2010) examination of procrastination, who analyzed 878 self-reported tasks recorded by 29 R&D engineers. For example, whereas Claessen et al. exclusively used self-report data, we also rely on observed behavior. Using a multilevel approach, our individual difference, self-regulatory and self-report workload variables are level two, based on 49 individuals, while the remaining task environment variables and observed workload are level 1, based on 1370 observed case characteristics.

For 49 level 2 participants, a single-tailed power analysis indicates a 67% chance of detecting as significant correlates of 0.30. Of note, the subsequent replication of these level 2 analyses with American arbitrators increases the statistical power to over 99%. Furthermore, as per Scherbaum and Ferreter (2009), these are extremely favorable conditions for multilevel modeling, matching their best-case scenario, which was 40 level 2 participants with an average sample size of 30 for level 1 (i.e., 1200 cases). Scherbaum and Ferreter estimate statistical power for this condition based on a medium effect is 95%.

**FIGURE 1:**
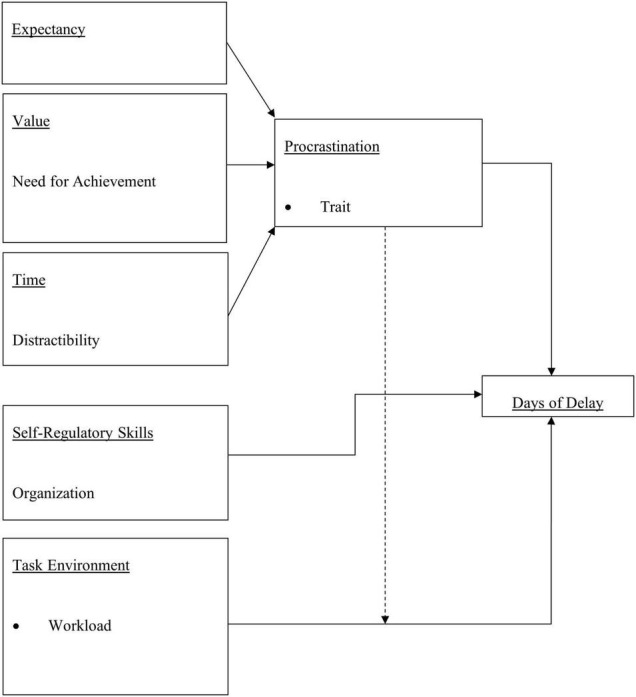
Relationship among individual difference motivational variables, self-regulatory skills, task environment and observed days of delay.

To assist in following the expected relationships, we summarize the variables graphically in Figure 1, with the dotted line indicated the expected interaction between procrastination and grievance complexity. We discuss the methodology to examine each of these relationships in turn.

### Participants (Level 2)

Our target population of Canadian arbitrators who are members of the NAA is relatively small, approximately 60–70 people depending on the exact year. From this group, 49 agreed to participate, a response rate of approximately 75%. This response rate is largely due, as mentioned, to two of the authors being active within the NAA. Also, these respondents represent a high proportion of the entire labor arbitration “business” as it is highly concentrated. Between 31 and 35% of all Canadian cases are decided by NAA members (Trudeau, 2002). Average age was approximately 46–50, 75% were men, and 80% were married. The average years of arbitration practice was 16–20, though most were NAA members for only 6 to 10 years.

Participants responded to over 200 questions, either through an online survey or identical hardcopy paper versions handed out during the NAA Annual Meeting. This approach was necessary to ensure coverage across all participants, though the majority of arbitrators responded through the online version. There were no cases in which a participant responded to both versions of the survey. Means, standard deviations and reliabilities of the measures are all reported in Table 1. To provide ensure domain coverage, the individual difference variables were typically operationalized in several ways, all at the trait level. Procrastination was assessed through two scales, the nine item Irrational Procrastination Scale (Steel, 2010; e.g., “I delay tasks beyond what is reasonable”) and a three-item content valid procrastination scale specific for arbitration (e.g., “The quality of my arbitration decisions is impaired by my procrastination”). The measurement of expectancy was through the Work Self-Efficacy Scale (Speier and Frese, 1997; e.g., “If I am in trouble, I can think of a solution”). The measurement of value was assessed at the trait level by three scales. Energy was measured by the Lack of Energy Scale (Kuhl and Fuhrmann, 1998; e.g., “If work is distasteful or boring, I have to force myself to get going”), need for achievement was measured by the Personality Research Form (Jackson, 1984; e.g., “I enjoy difficult work”), and task aversiveness for arbitration writing was measured by a content valid three-item scale (e.g., “I enjoy writing arbitration decisions” – reverse scored). Finally, sensitivity to delay was measured by the Attentional Distractibility Scale (Kuhl and Fuhrmann, 1998; e.g., “My mind wanders when I try to concentrate”) and the Susceptibility to Temptation Scale (Steel, 2010; “It takes a lot for me to delay gratification”).

**TABLE 1:** Descriptive statistics and Pearson correlations among personality and self-regulatory variables for Canadian arbitrators.

	Mean	Std.	1	2	3	4	5	6	7	8	9	10
**Procrastination**												
1 Trait Procrastination	2.33	0.63	*0*.*82*									
2 Arbitration Procrast.	2.28	0.78	0.52**	*0*.*73*								
**Expectancy Variables**												
3 Self-Efficacy	4.08	0.47	−0.34*	−0.38**	*0*.*85*							
**Value Variables**												
4 Task Aversiveness	2.18	0.82	0.33*	0.14	–0.04	*0*.*71*						
5 Lack of Energy	2.09	0.62	0.58**	0.31*	–0.11	0.34*	*0*.*82*					
6 Need for Achievement	3.58	0.54	−0.30*	–0.38	0.60**	–0.07	−0.31*	*0*.*84*				
**Temporal Variables**												
7 Distractibility	1.98	0.57	0.31*	0.20	–0.27	0.20	0.62**	−0.43**	*0*.*80*			
8 Susceptibility to Tempt.	2.17	0.61	0.69**	0.43**	–0.20	0.43**	0.65**	−0.38**	0.37**	*0*.*83*		
**Self-Regulatory Skills**												
9 Organization	4.03	0.62	−0.61*	−0.31*	0.44**	–0.22	−0.29*	0.34*	–0.16	–0.11	*0*.*51*	
10 Multitasking	2.86	0.64	0.36*	0.28	0.08	0.15	0.33*	0.07	0.10	0.44**	–0.01	*0*.*77*

N = 49. Cronbach Alpha italicized along the diagonal. *p < 0.05, **p < 0.01.

For the self-regulatory variables, we devised scales relevant for the arbitration practice based on inputs from two authors of the study who are part of the arbitration community. Organization was measured by a three-item scale (e.g., “I am very methodical in the way I approach my arbitration practice”). Multitasking was assessed with a 12-item scale (e.g., “I multitask,” “I write in short bursts between other non-writing activities”).

For busyness, we had the arbitrators retrospectively estimate work and personal commitments across separate life domains for that period. On a five-point scale, ranging from “Not at all” to “Extremely,” they indicated how involved they were in the following areas: Arbitrator selection, processes and evaluations; Mentoring other arbitrators; Conference preparation; Non-arbitration work; Physical fitness and recreation; Caring for children or grandchildren; Elder care for relatives; and Charity/community events/volunteer work. These life domains were selected based on a pilot study where five NAA practitioners were interviewed about activities that could impact the speed at which they write decisions. A self-report Workload item was devised by summing responses across all life domains.

### Task Environment (Level 1)

By law, almost all arbitrators in Canada must file their decisions with a Ministry of Labour or an equivalent body and these decisions are publicly available. We acquired all cases produced by our arbitrator sample over a 3-year period (2003--2005, inclusive). Three years’ worth of cases enabled us to develop an accurate portrait of the ‘‘normal’’ workflow of any arbitrator. The comprehensive data set of cases is a significant advantage of doing research in the Canadian setting. By contrast, American arbitration decisions are not routinely filed and are considered to be private documents^3^.

From the original group of 49 participating arbitrators, 41 were practicing during our sample period or provided hearing dates with which to measure arbitration delay. In total, LexisNexis^®^ *Quicklaw*™ Research Service provided full-text retrieval of all 1,370 cases, for an average of 33.4 cases per arbitrator. This number reduced to 1,204 cases from 40 arbitrators after eliminating panel decisions. Notably, this figure compares favorably with the 350 cases analyzed by Thornicroft (1995), the 600 cases by Ponak et al. (1996) or the 800 cases by Barnacle (1991).

Our coding adopted the approach of Ponak et al. (1996). The dependent variable, Decision Time, is the elapsed time between the date of the final hearing and the issuance of the award. Decision Time is an unobtrusive and concrete measure, as arbitrators routinely include this information without any notion that researchers will make use of it. We found that the average Decision Time was 61 days, comparable to the average of 93 days reported in the United States (Federal Mediation and Conciliation Service, 2011). However, with a range from same day to 1639 days per case or an average delay of 13 to 718 days per arbitrator, the positive skew was substantial. Accordingly, for comparisons with level 2 variables, we used a logarithmic transformation, reducing skew from 5.92 to 1.48 but still correlating with the untransformed scale at 0.79.

In addition, we distinguished regular and expedited cases and coded for grievance complexity. Complexity was assessed in terms of page length and time span of hearing. However, after standardizing these two variables, they formed a scale with a Cronbach’s Alpha of just 0.52, suggesting separate analyses for each. Finally, workload was operationalized as the publicly available written arbitration decisions the arbitrator released in the given year.

## Results

Means, standard deviations and reliability among the self-reported procrastination and self-regulatory variables, where applicable, are reported in Table 1. Compared with other adult (40 + years) employes, arbitrators are over a full standard deviation higher in Self-Efficacy (Speier and Frese, 1997) and Need for Achievement (Jackson, 1984) but lower in Trait Procrastination (Steel, 2010). Range restriction was not a concern, with standard deviations being no lower than 75% of population estimates. We used Harman’s Single-Factor Test to evaluate the extent of common method variance after grouping items into three packets. The items were divided into packets because using all of them simultaneously resulted in a non-positive definite matrix. The single factor accounted for substantially less than 50% of the variance, which is the threshold used to indicate common method concerns, with estimates of 13, 28, and 26% of the variance across item subsets. All scales show good reliability with the exception of Organization, which has an alpha of 0.51 but it is retained in the analyses as it still achieves a correlation of −0.61 with procrastination.

We investigated the relationship that Decision Time has with the level 2 variables using Weighted Least Squares (WLS) regression. The number of cases upon which to estimate Decision Time varied from 1 to 101 and consequently we give more weight to arbitrators with more cases as they will provide a better estimate of delay. The correlation between number of cases and procrastination itself was non-significant (*r* = −0.18, *p* = 0.26). WLS regression results are reported in Table 2. Procrastination had a strong relationship with observed delay, accounting for 23% of the variance in this measure. Arbitration Procrastination, despite being specific to the situation, predicted slightly less variance, 17%. Trait Procrastination predicts arbitrator delay, supporting Hypothesis 1.1.

**TABLE 2:** WLS bivariate regression of arbitrator decision time (log transformed) with personality and self-regulatory variables.

	*R*	*R* ^2^	*B*	*p*
**Procrastination**				
Trait Procrastination	0.40	0.16	0.41	0.011
Arbitration Procrastination	0.38	0.15	0.35	0.015
**Expectancy Variables**				
Self-Efficacy	0.04	0.00	−0.05	0.800
**Value Variables**				
Task Aversiveness	0.48	0.23	0.37	0.002
Lack of Energy	0.32	0.10	0.38	0.046
Need for Achievement	0.32	0.10	−0.36	0.046
**Temporal Variables**				
Distractibility	0.35	0.12	0.40	0.026
Susceptibility to Temptation	0.40	0.16	0.46	0.012
**Self-Regulatory Skills**				
Organization	0.36	0.13	−0.33	0.023
Multitasking	0.48	0.23	0.45	0.002
**Workload**				
Observed Workload	0.03	0.00	0.00	0.842
Self-Report Workload	0.42	0.17	0.50	0.008

*N = 40.*

We then investigated procrastination’s relationship with Expectancy, Value and Sensitivity to Time related trait variables. As shown in Table 1, all the variables correlate as expected with Trait Procrastination. Arbitration Procrastination shows a similar relationship, though Task Aversiveness and Distractibility slip below statistical significance. Table 3 provides a hierarchical regression of dispositional and self-regulatory variables predicting Trait Procrastination. Every step incrementally predicts, and 57% (50% adjusted *R*^2^) of the variance is accounted for with the dispositional variables alone, supporting Hypothesis 1.2. To investigate Hypothesis 1.3 (whether Trait Procrastination mediates the relationship between Expectancy, Value and Sensitivity to Time and Decision Time), we conducted a series of Sobel tests for mediation. Taking the strongest relationship between Decision Time at each hierarchical step, as per Table 2, we tested the mediation effects of Procrastination for Self-Efficacy, Lack of Energy and Susceptibility to Temptation using Preacher and Leonardelli (2015) interactive calculation tool. While regression weights and standard errors for Path A were obtained via linear regression, for Path B these were generated via WLS regression (as per Table 2), a methodology that does not accommodate alternative bootstrapping techniques sometimes employed for mediation based on smaller sample sizes. Still, in all cases there was significant and similar levels of mediation for Procrastination, with the Sobel test identical to the first decimal point (*p* = 0.04) across all three analyses.

**TABLE 3:** Hierarchical regression analysis predicting procrastination with dispositional and self-regulatory variables.

Step and predictor variable	*B*	*SEB*	*R* ^2^	Δ *R*^2^	*p*
1 Expectancy			0.11	0.11	0.021
**Self-Efficacy**	–0.45	0.19			
2 Value			0.43	0.32	<0.001
Self-Efficacy	–0.41	0.20			
**Task Aversiveness**	0.12	0.09			
**Lack of Energy**	0.52	0.13			
**Need for Ach.**	0.06	0.18			
3 Sensitivity to Time			0.57	0.14	0.003
Self-Efficacy	–0.41	0.18			
Task Aversiveness	0.02	0.09			
Lack of Energy	0.30	0.17			
Need for Ach.	0.16	0.17			
**Distractibility**	–0.10	0.15			
**Susceptibility to Temptation**	0.53	0.15			
4 Self-Regulatory Skills			0.73	0.16	<0.001
Self-Efficacy	–0.15	0.16			
Task Aversiveness	–0.04	0.07			
Lack of Energy	0.11	0.14			
Need for Ach.	0.19	0.14			
Distractibility	0.02	0.13			
Susceptibility to Temptation	0.54	0.14			
**Organization**	–0.48	0.10			
**Multitasking**	0.09	0.10			

*N = 48. Bolded variables are entered during that step.*

We then tested the relationship Decision Time has with the Expectancy, Value and Sensitivity to Time related trait variables, that is Hypotheses 2.1 through 4.2. As expected, being more distal to Decision Time than procrastination (procrastination being just one factor that can increase delay), these relationships have on average roughly half the effect size, with an average *R* of 0.28 compared to procrastination’s 0.45 (Table 2). Regarding Expectancy (Hypothesis 2.1), received no support (*p* = 0.90). On the other hand, Hypotheses 3.1 and 3.2 regarding Value were supported. Task Aversiveness (*p* = 0.02) and Lack of Energy (*p* = 0.02) were significant. Also, Need for Achievement (Hypothesis 3.3) was trending in the expected direction (*B* = −0.32) and statistically significant for a one-tailed test (*p* = 0.09). Similarly, Sensitivity to Time’s Hypothesis 4.2 for Susceptibility to Temptation was significant (*p* = 0.01) and Distractibility (Hypothesis 4.1) trending in the expected direction (*B* = 0.30) as well as borderline statistically significant at one-tailed (*p* = 0.10). Hypotheses 5.1 and 5.2 are addressed in the subsequent dataset.

Concerning self-regulatory skills, Table 1 considers their relationship with Procrastination and Table 2 with Decision Time. Organization and Multitasking are strongly related to both Procrastination and Decision Time, supporting Hypotheses 6.1, 6.2, 7.1, and 7.2. Similarly supportive, as set out in Table 3, Organization and Multitasking incrementally predict Trait Procrastination, adding 16% additional variance for a total of 73% (67% adjusted *R*^2^), with most of the variance provided by Organization. As per Table 1, Organization is negatively associated with Trait and Arbitrator Procrastination. Regarding Multitasking, it is associated with Trait Procrastination, though drops below significance for Arbitrator Procrastination. Using Preacher and Leonardelli (2015) interactive calculation tool as previously reviewed, Hypothesis 6.3 was confirmed: the Sobel test was significant (*p* = 0.03), and Organization mediates the relationship between Procrastination and Decision Time. Also, as per Hypothesis 7.3, Procrastination is associated with Susceptibility to Temptation, indicating as expected that the work strategy is typically detrimental and likely environmentally cued rather than autonomously pursued. Consistent with Hypothesis 7.4, Multitasking mediated the relationship between Susceptibility to Temptation and Procrastination (*p* = 0.04).

Bivariate correlations of Workload, both observed (*r* = 0.17, *p* = 0.30) and self-reported (*r* = −0.12, *p* = 0.40), with procrastination were not significant. Table 2 indicates that while Observed Workload was not related to Decision Time, there was a strong positive relationship between Self-Report Workload and Decision Time, supporting Hypothesis 9.1. Table 4 investigates this relationship further. Though all life domains have comparable mean levels of involvement, two accounted for most of the variance in Decision Time: (1) Arbitrator selection, processes and evaluations, and (2) Non-arbitration work. Consequently, this appears to be a form of role strain, such as role overload or intra-role conflict (Pearlin, 1989; Tubre and Collins, 2000). In other words, when there are several work tasks to pursue of similar importance or subject matter, interference among these tasks has the potential to increase, where one takes the place of another.

**TABLE 4:** Mean self-report workloads and WLS regression analysis predicting arbitrator delay with self-report workloads.

Predictor variable	*Mean*	*B*	*SEB*	*p*
Arbitrator selection, processes and evaluations	1.51	0.256	0.106	0.022
Mentoring other arbitrators	2.18	–0.273	0.154	0.087
Conference preparation	2.27	0.021	0.122	0.865
Non-arbitration work	2.07	0.175	0.087	0.055
Physical fitness and recreation	3.40	0.098	0.120	0.420
Caring for children or grandchildren	2.72	–0.001	0.071	0.989
Elder care for relatives	1.70	0.109	0.080	0.184
Charity/community events/volunteer work	2.07	0.017	0.083	0.844
Other hobbies	2.44	0.046	0.086	0.601

*N = 39. R^2^ = 0.47, Adj. R^2^ = 0.30, p = 0.017.*

### Multilevel Modeling

Because our data have a multilevel structure (cases nested within individuals), all analyses involving case or task characteristics were conducted using multilevel modeling using the *R* (version 4.1.1) package “nlme” (version 3.1–153). Like other widely used programs, nlme uses random-coefficient modeling for multilevel data and performs appropriate adjustments to the analytical procedures to account for nested data. Our complexity variables of Decision Length and Time Span of Hearing as well as Procrastination were centered prior to forming their cross-level interaction. Total observations were 1204 cases (level 1) from 40 arbitrators (level 2).

Descriptive statistics and a correlation matrix of our data are presented in Table 5. Table 6 presents the results of our multilevel analysis predicting days of delay. Initially, we tested a random intercept null model, where each arbitrator gets their own intercept, using it to calculate the Intraclass Correlation Coefficient (ICC). Approximately 66% of the variance is at level 2 or the arbitrator level, justifying taking a multilevel approach and supporting that individual-differences are a major contribution to delay.

**TABLE 5:** Descriptive statistics and Pearson correlations among variables for multilevel analysis.

	*Mean*	*SD*	1	2	3	4	5
**Level-One Variables**							
1 Days of delay	43.54	77.89	−				
2 Observed Workload	21.66	43.32	–0.01	−			
3 Expedited	0.09	0.28	−0.09**	–0.01	−		
4 Decision Length	10.68	9.53	0.24**	−0.06*	−0.10**	−	
5 Time Span	46.90	129.11	0.37**	–0.01	−0.07**	0.35**	−
**Level-Two Variable**							
6 Procrastination	0.00	1.00	0.13**	0.11**	–0.01	−0.09**	0.00

Level-1 observations, N = 1204; Level-2 observations, N = 40. *p < 0.05, **p < 0.01.

**TABLE 6:** Multilevel modeling results predicting decision time (days of delay).

	Value	Std. Error	*t*-score	*p*-value
**Level-One Coefficients**				
Constant	44.77	14.92	3.00	0.0028
Observed Workload	–0.07	0.13	–0.54	0.5900
Expedited	–20.54	6.87	–3.99	0.0000
Decision Length	1.19	0.23	5.25	0.0028
Time Span (standardized)	22.66	2.01	11.25	0.0000
**Level-Two Coefficient**				
Procrastination (standardized)	28.29	13.73	2.06	0.0463
**Cross-Level Interaction**				
Time Span × Procrastination	11.41	2.72	4.19	0.0000

*Level-1 observations, N = 1204; Level-2 observations, N = 40.*

The results reconfirm our previous analysis; Observed Workload is not related to delay. They also re-confirm that Trait Procrastination is significantly associated with the number of days it takes to render a decision (i.e., Hypothesis 1.1). The coefficient indicates that for each standard deviation increase in procrastination, elapsed time from hearing to decision increased by 28.29 days (*t* = 2.06, *p* = 0.046). Having a similar effect but in the opposite direction is Expedited cases, as per Hypothesis 10.1. Expediting a case *reduces* Decision Time by an average of 20.54 days (*t* = −2.99, *p* < 0.01). In other words, it would be preferable to select a low procrastination arbitrator without expediting than a high one with. Using the means from Table 5 with the coefficients from Table 6 (with the exception of Time Span, which being standardized has a mean of zero), we can calculate the impact of procrastination on delay. Keeping all other variables constant at their average, arbitrators one standard deviation above on procrastination take 82.94 days while those one standard deviation below take 26.38 days.

Finally, grievance complexity as measured by Decision Length increased Decision Time, with each written page adding 1.19 days (*t* = 5.25, *p* < 0.001). Similarly, our other complexity measure, the Time Span of Hearing Days, also significantly contributed to delay (*t* = 11.25, *p* < 0.001), which is somewhat harder to interpret as it follows a Pareto distribution with approximately half the cases (643 or 53.4%) at the lowest value or 1. Still, as the time span increases, so does decision time, supporting Hypothesis 11.1. For both these complexity measures, we detected an almost identical interaction with procrastination: for Decision Length (coefficient = 6.948, *t* = 3.67, *p* < 0.001) and for Time Span of Hearing Days (coefficient = 11.41, *t* = 4.19, *p* < 0.001). Given their redundancy, we incorporate just the interaction effect for Time Span of Hearing Days in Table 6 (though both analysis scripts are available in our OSF folder). To illustrate the interaction, we use reghelper’s (version 1.0.2) “nlme” option to run a Simple Slopes analysis in *R*. The simple slope for time span of hearing was positive and strong (coefficient = 34.07, *t* = 10.41, *p* < 0.001) for those who were one standard deviation above the mean in procrastination, but the simple slope for time span of hearing was a third the size (coefficient = 11.25, *t* = 3.22, *p* = 0.001) for those who were one standard deviation below the mean in procrastination, supporting Hypothesis 11.2. In other words, those low in procrastination pursued tasks at nearly the same pace regardless of the underlying complexity. Those high in procrastination increasingly dillydallied the more complicated tasks became.

## Study Two: American Arbitrators

Following up on the Canadian Arbitrators from Study One, we replicated as well as extended our study with a larger group of American Arbitrators. As mentioned, the task related variables were unavailable for this group as was the associated surreptitious measure of observed delay (i.e., unlike for Canada, this information is not part of the public record). However, we were able to assess the majority of the key self-report variables, including: procrastination, self-efficacy, task aversiveness, lack of energy, distractibility, work load/busyness, and multitasking. To address Hypotheses 5 and 8, we made space for two relevant variables, Pacing Style and Perfectionism, by swapping out the items related to Organization and Need for Achievement (i.e., the reliability of the former was marginal, and the latter was borderline significant).

## Materials and Methods

### Participants

Of the approximately 600 United States members of the NAA, we obtained responses from 195, or 35% of the total population. Average age reported was 70 to 79 by 51% of the respondents, 72.3% were men and 27.7% were women, 84.6% were married with 2% reporting never married, 95.4% reported having an advanced graduate and/or law degree, and 71.8% hearing arbitration cases for 26 years or more. This represents a well-established and experienced group of arbitrators.

### Measures

Measures employed were largely a subset of Study One’s, specifically those scales dealing with: procrastination, self-efficacy, task aversiveness, lack of energy, distractibility, and multitasking. Reference Study One for details. We also made one refinement and two additions. Consistent with Study One, we assessed self-report workload or busyness but this time used a more standardized scale, the Martin and Park Environmental Demands (MPED) Questionnaire (Martin and Park, 2003). Its six-item scale asks questions such as “I am very busy during an average day.” We also assessed Pacing Style using the nine-item Pacing Action Categories of Effort Distribution (PACED). It consists of three scales: *Deadlines* (completing work just before the due date; e.g., “I generally do not work until there is time pressure from an approaching deadline”), *U-Shaped* (completing work mostly at the start and finish with a break in between; e.g., “The effort I put into projects is high at the start, low half-way through, and high again at the end”), and *Steady* (complete work at an even pace throughout; e.g., “I work in a slow, but steady, manner to complete tasks”). Finally, to measure perfectionism’s discrepancy dimension, we used three items from the Revised Almost Perfect Scale (Slaney et al., 2001), framed to assess other-focused rather than self-focused discrepancy, specifically: “Others are hardly ever satisfied with my performance,” “My best just never seems to be good enough for others,” and “My performance rarely measures up to other people’s standards.” As per Study One, we used Harman’s Single-Factor Test to evaluate common method variance, and found that the single factors accounted for 29, 14, and 17% of variance across item subsets.

## Results

Correlations and univariate statistics are reported in Table 7. Contrasting Table 2, for Canadian Arbitrators, with Table 7, for American Arbitrators, both appear to be largely identical. Means and standard deviation were largely duplicated, with mean trait levels of procrastination remaining low across both groups (i.e., 2.33 versus 2.17). Correlations also replicated, with the exception of Distractibility, which showed *increased* correlations in Study Two. For example, its correlation with Procrastination increased from 0.31 to 0.61. In all, the original correlation matrix for Study One appears robust and generalizable. Consequently, the associated hypotheses from Study One also found support here. Multitasking correlates with Procrastination and Susceptibility to Temptation (Hypotheses 7.2 and 7.3) as well as mediates between them at *p* < 0.01 (Hypothesis 7.4).

**TABLE 7:** Descriptive statistics and Pearson correlations among self-report variables for American arbitrators.

	Mean	Std.	1	2	3	4	5	6	7	8	9	10	11	12
**Procrastination**														
1 Trait Procrastination	2.17	0.65	*0*.*86*											
**Expectancy Variables**														
2 Self-Efficacy	4.02	0.51	−0.24**	*0*.*77*										
**Value Variables**														
3 Task Aversiveness	1.98	0.82	0.37**	−0.21**	*0*.*71*									
4 Lack of Energy	2.18	0.64	0.61**	−0.15*	0.36**	*0*.*83*								
**Temporal Variables**														
5 Distractibility	2.09	0.70	0.61**	−0.26**	0.44**	0.58	*0*.*90*							
6 Susceptibility to Tempt.	2.11	0.58	0.70**	–0.12	0.29**	0.74**	0.70**	*0*.*84*						
**Pacing Style**														
7 Deadline	1.97	0.73	0.70**	0.00	0.32**	0.50**	0.35**	0.49**	*0*.*64*					
8 U Shaped	2.16	0.88	0.36**	–0.06	0.17*	0.43**	0.37**	0.44**	0.34**	*0*.*80*				
9 Steady	3.83	0.78	−0.70**	0.20**	−0.38**	−0.47**	−0.41**	−0.58**	−0.53**	−0.43**	*0*.*76*			
**Self-Regulatory Skills**														
10 Multitasking	2.71	0.54	0.35**	0.07	0.20**	0.24**	0.22**	0.31**	0.32**	0.26**	−0.16*	*0*.*67*		
**Workload**														
11 Self-Report Workload	2.41	0.69	0.34**	0.05	0.14	0.26**	0.26**	0.31**	0.24**	0.20**	−0.23**	0.59**	*0*.*75*	
**Perfectionism**														
12 Discrepancy	1.48	0.54	0.17*	−0.28**	0.16*	0.29**	0.24**	0.22**	0.00	0.34**	–0.10	0.05	0.02	*0*.*70*

N = 185 to 194 (due to pairwise deletion for missing responses). Cronbach Alpha italicized along the diagonal. *p < 0.05, **p < 0.01.

Nominally, the correlation between procrastination and deadline pacing style is 0.70. However, after disattentuating due to reliability, the corrected correlation increases to 0.94, indicating that they are interchangeable, supporting Hypothesis 8.1. The correlation with the u-shaped pacing style was much lower, at 0.36 or 0.43 corrected. The steady pacing style was −0.70 or −0.87 corrected. Though there can be times when people delay rationally, as in the u-shaped pacing style, as people reserve their work *exclusively* to the end, it strongly reflects procrastination. Also of interest is that busyness (self-report workload) and multi-tasking correlate positively with procrastination and with each other (*r* = 0.59). Notably, Malkoc and Tonietto (2019) would classify all these three variables as part of an activity maximization strategy, where to cope with our busyness we rely on deadlines and multitasking. In contrast, an outcome maximization strategy employs instead a steady pacing style.

Partially duplicating Table 3, the multiple regression prediction of procrastination with Canadian arbitrators, we have Table 8 for American arbitrators. Again, the results are largely the same, with a *R*^2^ of 0.57 after step 3 for Canadian arbitrators and a *R*^2^ of 0.56 after step 3 for American arbitrators, with a parallel pattern of beta weights. Hypothesis 1.2 is re-confirmed. Table 8 diverges in that it does not include the variables Organization or Need for Achievement, but otherwise the pattern of beta weights remains similar.

**TABLE 8:** Hierarchical regression analysis predicting procrastination with dispositional and self-regulatory variables for American arbitrators.

Step and predictor variable	*B*	*SEB*	*R* ^2^	Δ*R*^2^	*p*
1 Expectancy			0.06	0.06	0.001
**Self-Efficacy**	–0.32	0.01			
2 Value			0.44	0.38	< 0.001
Self-Efficacy	–0.18	0.08			
**Task Aversiveness**	0.11	0.05			
**Lack of Energy**	0.57	0.06			
3 Sensitivity to Time			0.56	0.12	< 0.001
Self-Efficacy	–0.15	0.07			
Task Aversiveness	0.08	0.05			
Lack of Energy	0.17	0.08			
**Distractibility**	0.17	0.07			
**Susceptibility to Temptation**	0.45	0.10			
4 Self-Regulatory Skills			0.58	0.02	0.01
Self-Efficacy	–0.16	0.07			
Task Aversiveness	0.06	0.05			
Lack of Energy	0.17	0.08			
Distractibility	0.17	0.07			
Susceptibility to Temptation	0.41	0.10			
**Multitasking**	0.18	0.07			

*N = 176. Bolded variables are entered during that step.*

Finally, we considered perfectionism’s connection to procrastination and whether it indeed is mediated. As per Table 7, the correlation between the two is 0.17 (*p* < 0.05), confirming Hypothesis 5.1. If we add perfectionism to Table 8’s series of regression analyses predicting procrastination, it increases *R*^2^ by a non-significant 0.001 (*p* = 0.280). Similar to Study One, we conducted a series of mediation analyses using the 2014 update of Preacher and Hayes (2004) Sobel test, with estimates based on 5,000 bootstrap resamples. The direct effect of perfectionism on procrastination for the following mediator models were: Self-Efficacy (0.137, *p* = 0.122), Distractibility (0.103, *p* = 0.664), Susceptibility to Temptation (0.027, *p* = 0.684), Lack of Energy (0.002, *p* = 0.973), Task Aversiveness (0.141, *p* = 0.085), and Multitasking (0.188, *p* = 0.023). With the exception of multitasking, every other variable’s mediation was sufficient to drive perfectionism’s direct effect to non-significance. As per Hypothesis 5.2, Sirois et al. (2017) suspicions and Xie et al. (2018) findings are borne out. Perfectionism is not directly related to procrastination.

## Discussion

Despite its ubiquity, procrastination has been almost exclusively studied with student samples and academic deadlines. We address this omission by studying observed delay in a critical area, the justice setting, where we are counseled that justice should not only be sure but also *swift*. Many nations embrace “speedy trial” principles, reflected in the Sixth Amendment to the United States Constitution and Section Eleven of the Canadian Charter of Rights and Freedoms. Here, we establish that procrastination appears to be endemic among the workplace, it is a major contributor to delay, it can be largely explained by Temporal Motivation Theory (Steel and König, 2006) and that procrastinators are especially susceptible to task difficulty or aversiveness. Procrastination, though unlikely to be eradicated, appears to be reduceable by addressing self-regulatory skills, such as increasing organizational skills and avoiding multitasking. There was little to no support for perfectionism being a cause of procrastination.

### The Prevalence of Procrastination

We studied arbitrators, who are both an ideal and a stringent test of personality and task effects on workplace procrastination. Arbitrators have high degrees of autonomy, meaning that personality effects should not be erased by high situational strength. The nature of their work provides a difficult to obtain criterion: an objective and valid measure of task delay, which is a key indicator of performance for arbitrators. It is stringent because although arbitrators have opportunities to delay, they have strong motivations not to and are carefully selected by both management and union representatives. As Domke and Edmonson (2003) underscore “The arbitrator is a decisive element any arbitration. The success or failure of arbitration will largely depend on them” (p. 24). Furthermore, given that arbitrators in our sample had practiced for a number of decades on average, there was ample time for less competent arbitrators or chronic procrastinators to self-select themselves out of the profession (Schneider, 1987). Indeed, average levels of procrastination, which are over 1.5 standard deviations lower than the average population (Steel, 2010), and other related traits indicate they are a group who should procrastinate remarkably little. And yet we still detected a substantive effect.

Given that procrastination made a substantive impact on the performance of this rarefied group of highly trained and motivated arbitrators, procrastination should be a significant factor in most autonomous work situations. Epidemiological work by Steel and Ferrari (2013) indicates that procrastination is indeed a global phenomenon with significant levels in most demographic categories. Supporting this conclusion is research showing procrastination substantively occurring in settings ranging from academic publication (Lay, 1987; Ackerman and Gross, 2007) to unemployment search (Lay and Brokenshire, 1997; van Hooft et al., 2005).

### The Importance of Procrastination

Since procrastination is putting off despite expecting to be worse off, it is naturally detrimental. Only its negative impact varies, tending to intensify the higher the stakes (Steel, 2011). As mentioned, timeliness is a critical performance dimension in the justice field in general and for arbitrators specifically and, as expected, trait procrastination was a major predictor of Decision Time. Arbitrators one standard deviation above the mean in terms of procrastination took over twice as long as arbitrators one standard deviation below. The effect of procrastination is virtually identical, though opposite in direction, to that of expedited cases, which represent the combined effect of several procedural interventions designed to speed task completion (e.g., allowing arbitrators to issue non-precedent setting awards). Similarly, procrastination’s effect on Decision Time was comparable to the effect of workload, which was the combined effect of other personal and work commitments. In short, personality effects on Decision Time were as strong as environmental procedures or external obligations.

Still, it is worth stressing that the procedural interventions were successful in reducing delay (i.e., expedited cases). If organizations find timeliness a problem, it can be partially ameliorated through well-designed processes. As Heath and Anderson (2010) note while reviewing external causes of undue delays, “supportive social scaffolding enables us to keep procrastination in check” (p. 233). For example, elements that speed task completion include the establishment of social norms, environmental cues and reminders, public and specific implementation intentions, and institutionally enforced rewards and repercussions (Steel, 2011). However, given that the practice of job design has generally drawn on mechanistic rather than motivational models, this avenue appears typically unexploited (Campion et al., 2005).

The importance of procrastination should be of more surprise to those who have adopted a rational model of human behavior, often associated with neo-classical economics (Posner, 1998; Ashraf et al., 2005). According to economic theory, which is essentially Expectancy × Value formulations, procrastination should not exist, being an irrational delay, or at least not have a substantive impact. Reflecting the lack of field research on the topic, as per Berg and Gigerenzer (2010), information of the sort presented is exceedingly rare, stating that “Notably missing is investigation of whether people who deviate from axiomatic rationality face economically significant losses” (p. 133) and “the normative interpretation of deviations as mistakes does not follow from an empirical investigation linking deviations to negative outcomes” (p. 150). However, the results here are consistent with the meta-analytic research showing a reliably negative relationship between performance and procrastination (Steel, 2007) as well as research connecting procrastination to diminished health and reduced financial success (e.g., Mehrabian, 2000; Elliot, 2002; Sirois et al., 2003). When people report that they tend to put off despite expecting to be worse off, their expectations are often borne out.

### Explaining Procrastination

There are several competing theories of procrastination, especially that procrastination is caused by perfectionism (e.g., Egan et al., 2011; Mohamadi et al., 2012). Here, we conducted an explicit test of Temporal Motivation Theory (Steel and König, 2006), which decomposes procrastination into expectancy, value and sensitivity to time related variables. Together, these variables account for approximately 57% of the variance at the trait level and their effect on observed delay is indeed mediated by procrastination. Furthermore, as predicted by Temporal Motivation Theory and confirmed by the multilevel analysis, procrastinators were especially vulnerable to difficult and less enjoyable tasks, as operationalized by grievance complexity. Those low in procrastination were far less affected by aversive task characteristics while those high in procrastination increasingly tended to delay. Given that procrastination is associated with reduced self-efficacy, these results are also consistent with Beck and Schmidt (2012) experimental research on self-efficacy, resource allocation, and goal difficulty. The more difficult a task becomes, the fewer resources are allocated to it by those lacking self-efficacy. Similarly, Louro et al. (2007) argue that resource allocation is a function of goal proximity and the valence of goal relevant emotions. Taken together, we are starting to have a firm understanding of when as well as why procrastination occurs.

The results from our investigation into self-regulatory skills and perfectionism spanned the range of outcomes: positive, negative and neutral. Notably, the propensity to procrastinate was incrementally predicted by self-regulatory skills, particularly Organization. In the other direction, Multitasking is confirmed as a largely dysfunctional work strategy, associated with procrastination, longer delays and being susceptible to temptations. In total, trait and self-regulatory variables accounted for an impressive 73% of the variance in procrastination in Study One and 58% in Study Two (which did not assess Organization). This almost exactly duplicates Steel et al. (2018) finding; a few variables accounted for 74% of the variance in procrastination, drastically limiting the amount of residual variance left to predict and other variables’ potential unique impact, such as perfectionism. As stressed here as well as by Xie et al. (2018), perfectionism’s connection to procrastination accounts for no incremental variance after these mediating processes are taken into account.

Again, the results stress the need to include time related variables and longitudinal perspectives both in motivational theory and practice. For example, Pepper and Gore (2014), also using a Temporal Motivation Theory frame, found that the excessive application of Expectancy × Value formulations has resulted in largely ineffective compensation plans, where existing “long-term incentives are not an efficient way of motivating senior executives, irrespective of national culture” (p. 26). In short, there are considerable costs in employing an overly stripped down or simplified motivational model.

### Limitations and Future Research

This research underscores many of the same points recently made in the motivational field (Fishbach and Zhang, 2009; Mitchell et al., 2009; Lord et al., 2010), such as Beck and Schmidt (2012) who discuss how we should view “motivation as a process of decision-making over time” and that “goal difficulty is a critical determinant of motivational processes” (p. 206). Accordingly, future research should continue along these lines, looking at motivation dynamically. We consider here a potential research program that facilitates this pursuit, which also addresses inherent limitations with this study.

One of the limitations of this study comes from its strength, our realistic or NDM setting (Lipshitz et al., 2001; Klein, 2008). Our use of arbitrators enabled a rigorous, unobtrusive, temporal measure of the work performed. It also makes replication extraordinarily difficult, especially without collaboration from within the sample frame, that is, fellow arbitrators. Even with a collaborator (i.e., two of the authors are active within the arbitration community), acquiring participants while keeping the assessment battery brief enough and with sufficient face validity to ensure completion was a reoccurring issue. We struck a balance in assessment by using a combination of context specific as well as more general scales (e.g., as for procrastination). Despite this issue, we did manage to administer a large assessment battery of up to 200 items and were able to replicate many of the results in Study Two with a much larger sample. Still, for this type of multilevel design, Study One represents a large number of participants and cases (e.g., Claessens et al., 2010).

Finally, using archival case data limits the information available from these cases to what has been recorded. For example, we are unable to examine state level manifestations of expectancy, value and sensitivity to time that the arbitrators actually experience as they encounter each individual case. Accordingly, these issues are somewhat unavoidable, inherent to constraints that most field studies with measures of actual behavior encounter (Baumeister et al., 2007). However, that the available case information exists is itself notable. The arbitration cases themselves are part of the public record within the Canadian system but are only sporadically available in other countries, such as the United States. To our knowledge, there are no other comparable groups of professionals where people are performing essentially the same or easily comparable but measurable “slippery deadline” task frequently over potentially several years. However, there is a promising non-professional option to explore.

Steel and König (2006) recommend investigating motivation dynamically with courses taught through a computerized personal system of instruction (C-PSI). As they review, a C-PSI course allows: “hundreds of students simultaneously working toward completing a university course at their own pace, allowing choice and thus motivated behavior. Furthermore, progress is assessed at a large number of data points as the course is broken down into numerous assignments, all computer administered with completion precisely recorded. Similarly, a host of other observed and self-reported measures can be easily inserted into this framework” (p. 906–907). In short, we have a meaningful task, a “slippery deadline,” and precise start and completion dates. As per the present study, this is ideal since the tight connection between observed delay and procrastination means, *in the absence of an accompanied early start*, observed delay over several tasks can be taken as a reasonable proxy of procrastination despite the lack of a formal assessment of irrationality. Previous use of the venue by Steel et al. (2001) established that C-PSI courses are rife with procrastination and other forms of self-regulatory failure.

Furthermore, there has been rapid growth of Massive Open Online Courses (MOOCs), such as those being offered by MIT, Harvard and Berkeley (Martin, 2012). With enrollment in the hundreds of thousands, rather than the hundreds, it allows for an ongoing and detailed investigation of self-regulation. Partly due to our choice of sample, our battery of self-regulatory measures was limited and a few measurement issues arose after contextualizing it to an arbitration setting (e.g., the reliability for the self-regulatory measure of Organization was lower than expected). Though we could touch on the importance of self-regulation, proper examination of self-regulation requires deep examination, befitting a topic that consists of “multiple processes and components that interact in complex ways” (Boekaerts, 2010, p. 71). MOOCs appear ideal for this purpose, given that their sample size and number allow for continuous and varied exploration (Diver and Martinez, 2015).

The benefit of a more detailed examination would be to isolate key proximal skills that influence goal striving but are also trainable. As Steel and Klingsieck (2016) emphasize, procrastination interventions should not focus on the neurotic procrastinator, which comprises perhaps 10% of all procrastinators, but customized to individuals’ specific set of temptations, circumstances and target tasks. Since procrastination has separate expectancy, value and impulsiveness components, treatment should first proceed by identifying specific weaknesses in these areas and then targeting appropriate skills. For example, those specifically lacking self-efficacy will not necessarily respond to value interventions (e.g., interest enhancement) or impulsiveness interventions (e.g., attentional control). One size does not fit all. Online computerized courses will enable us to assess the interaction effect between prior need and the development of self-regulatory skills, rather easily if the self-regulatory interventions can also be administered as a C-PSI. If the massive and constantly renewing sample offered by MOOCs can be drawn upon, the dynamic study of motivation can be expected to advance quite rapidly. A particularly notable though early effort along these lines is Huang et al. (2021).

Finally, arbitrators represent some of the lowest scoring professions for procrastination, but even within this elite group procrastination had an impact. In today’s deadline strewn and temptation-soaked world, completely avoiding procrastination appears to be a rarity. As research such as Rinaldi et al. (2021) stress, this ubiquity reflects that the neurobiological roots of procrastination run through the brain’s executive function, which we all have. Though this is consistent with Temporal Motivation Theory (Steel, 2011), we would benefit by moving beyond summary variables (e.g., impulsivity) and take a more detailed approach. By identifying the exact neural mechanisms that are impaired or at least operating less than optimally, new diagnostics and interventions to minimize procrastination are likely to emerge.

## Conclusion

Members of the National Academy of Arbitrators – individuals at the pinnacle of their profession – are still substantively affected by procrastination. Having found procrastination in this venue, we likely will find it to exert a significant influence on any job where people can autonomously choose their work schedule, such as writers, entrepreneurs or independent salespeople as they all have considerable discretion over task completion. In particular, procrastination should become an increasing issue for senior management. For example, Spencer (1955) surveyed 950 company presidents and chief executives, finding that procrastination was the most troublesome problem reported and “It was also evident that personal procrastination was involved in scores of other problems they mentioned, even though the term was not used” (p. 83). Given procrastination’s broad manifestation and evident importance, we should not underestimate the temporal nature of work behavior, which is affected by both task and individual variables.

## Data Availability Statement

The original contributions presented in the study are included in the article/supplementary material, further inquiries can be directed to the corresponding author/s.

## Ethics Statement

The studies involving human participants were reviewed and approved by Haskayne School of Business, University of Calgary Conjoint Faculties Research Ethics Board. The patients/participants provided their written informed consent to participate in this study.

## Author Contributions

DT and AP have contributed equally to this work. All authors contributed to the article and approved the submitted version.

## Conflict of Interest

The authors declare that the research was conducted in the absence of any commercial or financial relationships that could be construed as a potential conflict of interest.

## Publisher’s Note

All claims expressed in this article are solely those of the authors and do not necessarily represent those of their affiliated organizations, or those of the publisher, the editors and the reviewers. Any product that may be evaluated in this article, or claim that may be made by its manufacturer, is not guaranteed or endorsed by the publisher.

## Footnotes

1 Some collective bargaining agreements have specified time limits for rendering a decision, but such time limits are routinely waived by the union and employer.

2 In Canada, from which one sample of our arbitrators are drawn, the law requires that all arbitration decisions be publicly reported and are available electronically.

3 Publicly reported labor arbitration decisions in the United States are not representative of the universe of labor arbitration decisions (Steiber et al., 1985). In contrast, since all labor arbitration decisions in Canada must be reported by law, our data set contains the universe of decisions of the arbitrators in our sample.
